# Effects of ethanol extract of *Ficus bengalensis* (bark) on inflammatory bowel disease

**DOI:** 10.4103/0253-7613.68420

**Published:** 2010-08

**Authors:** Manish Amerutlal Patel, Paras Kantibhai Patel, Manish B. Patel

**Affiliations:** Department of Pharmacology, C.K. Pithawala Institute of Pharmaceutical Sciences and Research, Surat - 395 007, Gujarat, India; 1 Shri Sarvajanik College of Pharmacy, Mahesana, Gujarat, India

**Keywords:** Bark, ethanolic extract, *Ficus bengalensis* (Moraceae), inflammatory bowel disease

## Abstract

**Objective:**

The present study was designed to evaluate the effects of ethanol extract of *Ficus bengalensis* Linn. bark (AEFB) on inflammatory bowel disease (IBD).

**Materials and Methods:**

Effects of AEFB were studied on 2,4,6-trinitrobenzenesulfonic acid (TNBS, 0.25 ml 120 mg/ml in 50% ethanol intrarectally, on first day only) induced IBD in rats. The effects of co-administration of prednisolone (2 mg/kg) and AEFB (250, 500 mg/kg) for 21 days were evaluated. Animals sacrificed at end of the experiment and various histopathological parameters like colon mucosal damage index (CMDI) and disease activity index (DAI) were assessed. In the colon homogenate malondialdehyde (MDA), myeloperoxidase (MPO), superoxide dismutase (SOD), and nitric oxide (NO) levels and in mesentery % mast cell protection was also measured.

**Results:**

Rats treated with only TNBS showed more score of CMDI and DAI, higher MDA, NO, MPO, and lower SOD activity as compared to the control group. Treatment with AEFB significantly declined both indices scores and decreased the MPO, MDA, NO, and increased the SOD activity. AEFB also increased the % mast cell protection compared to alone TNBS-treated animals.

**Conclusion:**

In our study, we found that AEFB has a significant protective effect in the IBD in rats that is comparable to that of prednisolone and may be because of the presence of flavonoids, terpenoids, and phenolic compounds.

## Introduction

Inflammatory bowel disease (IBD) encompasses many chronic, relapsing inflammatory disorders involving the gastrointestinal tract.[1] In IBD, the intestine (bowel) becomes inflamed, often causing recurring abdominal cramps and diarrhea. Among the pathological findings associated with IBD are increases in certain inflammatory mediators, signs of oxidative stress, a deranged colonic milieu, abnormal glycosaminoglycan content of the mucosa, increased intestinal permeability, increased sulphide production, and decreased methylation.[2] The available treatment choices have major limits owing to the associated adverse effects and compliance issues.[1] As a result, there is high prevalence of complementary and alternative medicines for treating the mentioned disease.

*Ficus bengalensis* Linn. (Family: Moraceae) is a reputed plant in ayurvedic medicine and commonly known as “banayan tree” in ayurvedic literature. Milky juice from stem, seeds, or fruits of the plant is applied externally in rheumatism and to the soles of feet when inflamed, internally used in dysentery and diarrhea.[3] All the parts of the plant have astringent, anti-inflammatory, antiarthritic, and antidiarrheal activities. The latex is useful in hemorrhage, diarrhea, and dysentery, as well as in hemorrhoid and inflammation.[4] Severe inflammation and diarrhea are the characteristics of IBD. So, with the light of the folkloric usage of the bark mainly in inflammation and diarrhea, this study was carried out to evaluate the efficacy of the ethanolic extract of *F. bengalensis* bark (AEFB) in IBD.

## Materials and Methods

### Plant Materials

The stem barks of *F. bengalensis* Linn. was collected from South Gujarat region. The plant was identified and authenticated by Dr. Minoo Parabia, Department of Bioscience, VNSGU, Gujarat, India (Voucher specimen number is HMG/0404/2007).

### Preparation of Plant Extract

The stem bark of the plant was air dried, reduced to coarse powder, macerated with ethanol for 48 h, filtered, and the filtrate was evaporated under reduce pressure to obtain dry extract. The extract was stored in cool and dry place and was used for pharmacological evaluation after suspended in 0.5% carboxyl methylcellulose (CMC) prior to administration. Various phytoconstituents qualitatively were determined in AEFB according to Wagner and Bladt.[5]

### Animals

Adult albino Wistar rats of either sex weighing between 200-250 g housed under standard conditions of temperature (22 ± 2°C), relative humidity (55 ± 5%), and light (12 h light/dark cycles) were used. They have been fed with standard pellet diet and water *ad libitum*. Animal were approved by the Institutional Animal Ethics Committee according to the regulation of Committee for the Purpose of Control and Supervision of Experiments on Animals.

### Acute Toxicity Testing

Albino Wistar rats of either sex weighing 200-250 g were used in the study. An acute oral toxicity study was performed as per Organisation for Economic Co-operation and Development (OECD)-423 guideline. Albino rats of either sex weighing 200-250 g selected by the random sampling technique were used in the study. Acute oral toxicity was performed as per OECD-423 guidelines. The animals were fasted overnight, provided only water, after which the drug AEFB was administered to the respective groups orally at the dose level of 5 mg/kg body weight and the groups, observed for 14 days. If mortality was observed in two or three animals, then the dose administered was assigned as a toxic dose. If mortality was observed in one animal, then the same dose was repeated again to confirm the toxic dose. If mortality was not observed, the procedure was repeated for further higher doses such as 50, 300, and 2000 mg/kg body weight. The animals were observed for toxic symptoms such as behavioral changes, locomotion, convulsions, and mortality for 72 h.

### Experimental Protocol

Experimental IBD was induced in animals by intrarectally administration of TNBS (2, 4, 6-trinitrobenzenesulfonic acid) according to Farmin *et al*, 1996.[6] The study comprised five different groups of six animals each as follows:

Control: Saline treated;

Model: TNBS (0.25 ml, 120 mg/ml in 50% ethanol, intrarectally) on first day only;

Prednisolone: Prednisolone (2 mg/kg, p.o.) treatment continued till 21^st^ day; followed by 1^st^ day treatment of TNBS;

AEFB (250 mg/kg): AEFB (250 mg/kg, p.o.) treatment continued till 21^st^ day; as that of prednisolone;

AEFB (500 mg/kg): AEFB (500 mg/kg, p.o.) treatment continued till 21^st^ day as above.

TNBS was delivered by a Teflon cannula (outside diameter 1.2 mm, inserted 8 cm) through the anus of each rat. Ethanol evokes an acute inflammatory response which resolves spontaneously after 1 week. Therefore, we preferred to include a saline-treated group as a negative control instead of an ethanol-treated group. On 22^nd^ day, animals were sacrificed by cervical dislocation and dissected open to remove GIT (from stomach to anus). GIT was flushed gently with saline and cut open. Then the colon mucosal damage index (CMDI) and the histopathological score i.e. disease activity index (DAI) were evaluated. Colon samples were taken for determinations of myeloperoxidase (MPO) and superoxide dismutase (SOD) activity, malondialdehyde (MDA), and nitric oxide (NO) content levels. Percentage protection of the mast cell degranulation in the mesentery of intestine of the rat was also measured.

### Assessment of the Colon Mucosal Damage Index

The colon segment taken 10 cm proximal to anus of the sacrificed rats was excised longitudinally, rinsed with saline buffer, and fixed on a wax block. Each colon was observed and evaluated by two independent observers. Macroscopic scoring was done and evaluated according to the formula of CMDI reported by Wei *et al*.,[7] briefly describe as follows: (1) 0-Normal mucosa, 1-Mild hyperemia, no erosion or ulcer on the mucosal surface, (2) 2-Moderate hyperemia, erosion appearing on the mucosal surface, (3) 3-Severe hyperemia, necrosis and ulcer on the mucosal surface with the major ulcerative area extending <40%, (4) 4-Severe hyperemia, necrosis, and ulcer on the mucosal surface with the major ulcerative area extending >40%.

### Assessment of Disease Activity Index

The colon tissue samples taken for histology were fixed overnight in 4% neutral buffered formalin, processed, sectioned (4µm thick), and stained with hematoxylin and eosin. Each colon sample was observed and evaluated by two independent observers. The histopathological score was assessed following modified model of Wei *et al*.[7] as follows: (1) the infiltration of acute inflammatory cells: 0-no, 1-mild increasing, 2-severe increasing; (2) the infiltration of chronic inflammatory cells: 0-no, 1-mild increasing, 2-severe increasing; (3) the deposition of fibrotin protein: 0-negative, 1-positive; (4) the submucosa edema: 0-no, 1-patchy-like, 2 fusion-like; (5) the epithelium necrosis: 0-no, 1-limiting, 2-widening; (6) the epithelium ulcer: 0-negative, 1-positive.

### Determination of the MPO, SOD activity and MDA, NO content in the colon

The colon sample was homogenized (50 g/L) in 50 mmol/L ice-cold potassium phosphate buffer (pH 6.0) containing 0.5% of hexadecyltrimethylammonium bromide. The homogenate was first frozen and thawed three times, and then centrifuged at 4000 rpm for 20 min at 4°C for the measurement of MPO activity. MPO, a marker of neutrophil migration was estimated by measuring H_2_O_2_-dependent oxidation of *o*-dianisidine.[8]

For the determination of SOD activity and MDA, NO contents, the colon sample was homogenized in ice-cold phosphate buffer saline (pH 7.4) and centrifuged at 3000 rpm for 10 min at 4°C. The clear supernatant was used for the assay of MDA which is the indicator of lipid peroxidation,[9] and the endogenous antioxidant enzyme SOD was estimated according to Misra and Fridovich.[10] NO was determined according to the method of Yamamoto *et al*.[11]

### % Mesenteric Mast Cell Protection

% Mast cell protection is suggested by a decrease in the degranulation of the mast cell. Mesentery of intestine obtained from the animals was placed in the Ringer Locke solution (NaCl 0.9%, KCI 0.042%, CaCl_2_ 0.024%, NaHCO_3_ 0.015%, and dextrose 0.1%). Then it was stained and fixed with the 4% formaldehyde containing 0.1% toluidine blue. % Mast cell protection was evaluated microscopically at 40X magnification.[12]

### Statistical Analysis

Data obtained from the animal experiments were expressed as the mean ± SEM of six observations. The statistical difference was evaluated by one-way ANOVA. Differences were accepted as statistically significant when *P*<0.05.

## Results

The yield of AEFB was 4.8% w/w, and preliminary qualitative phytochemical analysis showed that it contained glycosides, carbohydrates, flavonoids, triterpenoids, tannins, and phenolic compounds. No mortality and the sign of toxicity were observed at the dose of 2000 mg/kg.

### Inflammatory Changes in the Mucosa of Colon

The main parameters used for evaluating the degree of colonic inflammation in IBD were CMDI, DAI, and MPO activities. In this study, significant differences in CMDI, DAI, and MPO activities were found, when compared with that of control and model groups. In prednisolone and AEFB-treated groups, CMDI and DAI and MPO activities significantly decreased when compared with the model group [Table 1].

**Table 1:** Effects of ethanolic extract of *F. bengalensis* on CMDI, DAI, and MPO activities in the colon tissue of TNBS-induced IBD in rats

*Groups*	*CMDI*	*DAI*	*MPO (unit/mg of protein)*
Control	0.0 ± 0.0	0.7 ± 0.11	22 ± 0.9
Model	6.1 ± 0.31*	8.3 ± 0.2*	88 ± 2.5*
Prednisolone	1.4 ± 0.21#	3.2 ± 0.10#	38 ± 1.2#
AEFB (250 mg/kg)	2.3 ± 0.13#	4.1 ± 0.13#	52 ± 15#
AEFB (500 mg/kg)	1.7 ± 0.18#	2.4 ± 0.11#	49 ± 1.8#

Each value presented as mean ± SEM (n=6) (one-way ANOVA);

* When compared to the control group *P*<0.001;

# When compared to the model group *P*<0.001;

(CMDI: colonic mucosal damage index, DAI: disease activity index, MPO: myeloperoxidase).

### Oxidative Changes in the Colon

Severe oxidative stress induced by intrarectal administration of TNBS showed significant elevation of MDA and NO level and a significant decrease in the SOD activity compared to the control group. After treatment with prednisolone and AEFB, there was a significant decrease both in MDA and NO levels and an increase in the SOD activity when compared with the model group, showing that AEFB has antioxidant properties [Table 2].

**Table 2:** Effects of ethanolic extract of *F. bengalensis* on MDA and NO content, SOD activity and mast cell degranulation in TNBS induce inflammatory bowel disease in rats

*Groups*	*MDA (nmoles/mg of protein)*	*NO (nmoles/mg of protein)*	*SOD (U/min/mg of protein)*	*Mast cell degranulation (%protection)*
Control	17 ± 0.7	12 ± 0.5	0.89 ± 0.05	80 ± 1.5
Model	88 ± 2.1*	28 ± 1.1*	0.22 ± 0.03*	8 ± 0.5*
Prednisolone	34 ± 1.4^#^	14 ± 0.8^#^	0.78 ± 0.05^#^	62 ± 1.0^#^
AEFB (250 mg/kg)	53 ± 1.7^#^	18 ± 0.5^#^	0.73 ± 0.02^#^	48 ± 0.8^#^
AEFB (500 mg/kg)	40 ± 1.8^#^	15 ± 0.6^#^	0.61 ± 0.03^#^	59 ± 0.9^#^

* (MDA: Malondialdehyde, NO: Nitric oxide, SOD: Superoxide dismutase);

Each value presented as mean ± SEM (n=6) (one-way ANOVA);

Compared with the control group *P*<0.001;

Compared with the model group *P*<0.001

### Mesenteric Mast Cell Degranulation

In the animals treated with the TNBS (model group), a significant increase in the mast cell degranulation was observed compared with the control group. Treatment of the prednisolone and AEFB significantly decreased the mast cell degranulation compared to TNBS-treated animals [Table 2].

### Histopathological Changes in the Colon

**Figure 1:**
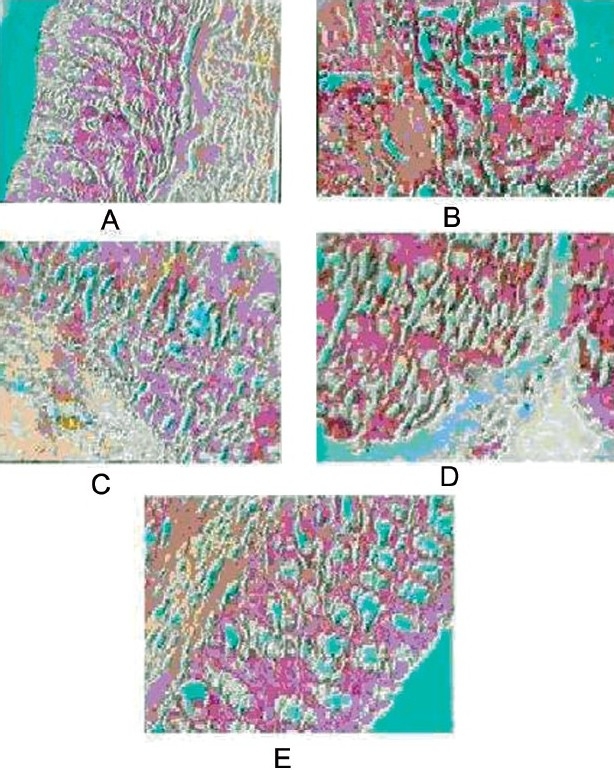
Effect of ethanolic extract of *F. bengalensis* on tissue histopathology in TNBS-induced IBD in rat. Control group: shows normal mucosa (A), model group: shows severe hyperplasia, ulcer appearing on the mucosal surface with the major ulcerative area extending >40% (B), prednisolone: shows moderate hyperplasia, no erosions appear on the mucosal surface (C), AEFB (250 mg/kg): shows mild hyperplasia and erosion appear on the mucosal surface (D), AEFB (500 mg/kg): mild hyperplasia, no ulceration on mucosa (E).

Histopathological analysis of the colon in TNBS-treated group clearly showed that there were severe hyperplasia, edema, infiltration of inflammatory cells, particularly nuetrophils and lymphocytes, necrosis, and ulcer on the mucosal surface, >40% showing the progression to severe disease [Figure 1b]. Animals treated with the prednisolone showed mild hyperplasia and edema, infiltration of inflammatory cells with decrease in normal mucosa, no necrosis and no ulceration [Figure 1c]. With the treatment with of AEFB, the progression of IBD was less prominent characterized by a decrease in hyperplasia and edema, and decline in the infiltration of the inflammatory cells. In addition, mild necrosis and ulceration were present in AEFB 250 mg/kg group [Figure 1d] but absent in AEFB 500 mg/kg group [Figure 1e].

## Discussion

TNBS is a hapten compound, and when it is bound with a substance of high molecular tissue proteins, it will turn into an antigen. It has been shown that it can elicit immunologic responses and induce generation of IBD.[13,14] This model shares many of the histopathological and clinical features of human IBD and is useful for the study of the etiopathogenesis of chronic colon inflammation as well as providing an inexpensive model suitable for assessing new therapeutic agents.

In the present study, IBD was introduced by intrarectal administration of TNBS in animals. The severity of colonic inflammation in developed disease was evaluated by measuring main parameters CMDI and DAI scores and MPO activity. A decrease in the progression of the disease pathogenesis following treatment with AEFB characterized by a significant decline in the score of CMDI and DAI was found compared to the model group. MPO is an enzyme found in the neutrophils and can be used as a quantitative index of inflammation in colonic tissue. The main pathological feature of IBD is transmural infiltration of polymorphonuclear neutrophils and MPO is released from these neutrophils.[15,16] Treatment with AEFB significantly decreased the level of MPO compared to the model group which shows that AEFB decreases the infiltration of the inflammatory cells which are responsible for the increasing progression of the disease condition.

Many studies have revealed that an increase of oxidative stress and iNOS activity was a notable feature of IBD, which resulted in a pathological cascade of free radical reactions and further yielding more oxidative free radicals. Failures of the endogenous antioxidant defense mechanisms promote formation of excessive free radicals and consequent tissue damage.[17] Parameters such as MDA, NO content, and SOD activity can be indicative of oxidative stress status of the disease. As observed in the study, the increase in MDA levels in the colon affected by the TNBS administration suggests enhanced lipid peroxidation which could be responsible for the tissue damage. Many studies have reported that an increased iNOS activity yielded more oxidative free radicals such as peroxynitrite (ONOO^–^) to impair the structure and function of the cells.[18] Excess of NO is responsible for the increasing of disease severity by increasing the vascular permeability and decreasing of the antioxidant defense mechanism by inhibiting the SOD enzyme.[19]

In the animal group treated with TNBS, an increase in the oxidative stress was observed indicated by the higher MDA and NO levels and as well as a decrease in the SOD activity which might be responsible for the tissue damage and development of inflammation. Animals treated with AEFB showed a significant decrease of both MDA and NO levels, and SOD activity was significantly increased confirming that AEFB decreased the tissue damage and inflammation suggesting its significant antioxidant property.

Mast cell degranulation causes mucus secretion, mucosal edema, increased gut permeability, and release of various inflammatory mediators which may be responsible for some of the signs and symptoms of IBD.[20] In the present study a significant rise in the mast cell degranulation was observed in the TNBS-treated animals, while in AEFB-treated animals, mast cell degranulation was significantly lower. This observation clearly indicates that AEFB provide the protection against the injury produced by inflammatory mediators released from the mast cell degranulation.

Prednisolone at a dose of 2 mg/kg and AEFB at doses of 250 mg/kg and 500 mg/kg provided the protection against TNBS-induced IBD in rats. It may be possible that AEFB produced this protection by the same mechanism as that of prednisolone i.e. inhibition of the infiltration of the inflammatory cells, antioxidant activity, and decreasing the synthesis of the inflammatory mediators.

Various phytochemicals like phenolic compounds and flavonoids present in AEFB may be responsible for its antioxidant effect. Flavonoids also have mast cell stabilizing property.[21,22] Terpenoids[23] have an anti-inflammatory effect, which may be responsible for the anti-inflammatory effect of AEFB in IBD. Due to the presence of above phytochemicals in the bark of *F. bengalensis* and the results obtained in this study, the bark of the title plant might be used for the treatment of IBD.
